# Can Multi-Parametric MR Based Approach Improve the Predictive Value of Pathological and Clinical Therapeutic Response in Breast Cancer Patients?

**DOI:** 10.3389/fonc.2018.00319

**Published:** 2018-08-15

**Authors:** Uma Sharma, Khushbu Agarwal, Rani G. Sah, Rajinder Parshad, Vurthaluru Seenu, Sandeep Mathur, Siddhartha D. Gupta, Naranamangalam R. Jagannathan

**Affiliations:** ^1^Department of NMR and MRI Facility, All India Institute of Medical Sciences, New Delhi, India; ^2^Department of Surgical Disciplines, All India Institute of Medical Sciences, New Delhi, India; ^3^Department of Pathology, All India Institute of Medical Sciences, New Delhi, India

**Keywords:** breast cancer, neoadjuvant chemotherapy, magnetic resonance spectroscopy, total choline, apparent diffusion coefficient, tumor volume, pathological response, clinical response

## Abstract

The potential of total choline (tCho), apparent diffusion coefficient (ADC) and tumor volume, both individually and in combination of all these three parameters (multi-parametric approach), was evaluated in predicting both pathological and clinical responses in 42 patients with locally advanced breast cancer (LABC) enrolled for neoadjuvant chemotherapy (NACT). Patients were sequentially examined by conventional MRI; diffusion weighted imaging and *in vivo* proton MR spectroscopy at 4 time points (pre-therapy, after I, II, and III NACT) at 1.5 T. Miller Payne grading system was used for pathological assessment of response. Of the 42 patients, 24 were pathological responders (pR) while 18 were pathological non-responders (pNR). Clinical response determination classified 26 patients as responders (cR) while 16 as non-responders (cNR). tCho and ADC showed significant changes after I NACT, however, MR measured tumor volume showed reduction only after II NACT both in pR and cR. After III NACT, the sensitivity to detect responders was highest for MR volume (83.3% for pR and 96.2% for cR) while the specificity was highest for ADC (76.5% for pR and 100% for cR). Combination of all three parameters exhibited lower sensitivity (66.7%) than MR volume for pR prediction, however, a moderate improvement was seen in specificity (58.8%). For the prediction of clinical response, multi-parametric approach showed 84.6% sensitivity with 100% specificity compared to MR volume (sensitivity 96.2%; specificity 80%). Kappa statistics demonstrated substantial agreement of clinical response with MR volume (*k* = 0.78) and with multi-parametric approach (*k* = 0.80) while moderate agreement was seen for tCho (*k* = 0.48) and ADC (*k* = 0.46). The values of k for tCho, MR volume and ADC were 0.31, 0.38, and 0.18 indicating fair, moderate, and slight agreement, respectively with pathological response. Moderate agreement (*k* = 0.44) was observed between clinical and pathological responses. Our study demonstrated that both tCho and ADC are strong predictors of assessment of early pathological and clinical responses. Multi-parametric approach yielded 100% specificity in predicting clinical response. Following III NACT, MR volume emerged as highly suitable predictor for both clinical and pathological assessments. PCA demonstrated separate clusters of pR vs. pNR and cR vs. cNR at post-therapy while with some overlap at pre-therapy.

## Introduction

Neoadjuvant chemotherapy (NACT) is the standard care of treatment for patients with locally advanced breast cancer (LABC) owing to large tumor size, location and the risk of disease dissemination (1, 2). Advantages of NACT include reduction in tumor size and early treatment of micro and distant metastasis, facilitating breast conservation surgery and improved clinical outcome (1, 2). However, chemotherapy has several side effects, complete pathologic response rates are low (10–31%) and overall response rates range from 69 to 100% (1, 2). Thus, it is essential to monitor the tumor's response sequentially after each NACT cycle to design patient tailored treatment. This would allow an early shift to alternative treatments and avoid toxicity of chemotherapy.

Conventionally, assessment of tumor response is carried out by physical examination and tumor size measurements by radiological techniques like X-ray mammography and ultrasound. However, these techniques do not accurately differentiate between chemotherapy-induced fibrosis and residual disease and both over- and under-estimation of tumor sizes have been documented (3–5). Further late manifestation of changes in tumor size has also been the limitation of these morphology based assessment methods (3–5).

Recently, the potential of various techniques that provide insight into chemotherapy induced changes in tumor physiology; vasculature and metabolic activity have been investigated. Positron emission tomography and single-photon emission computed tomography provide information on metabolic changes that occur due to therapy. However, their clinical utility is limited due to the use of repeated radiation (6).

These limitations have led to the exploration of various MR based techniques in the assessment of chemotherapy response (7–9). Being non-invasive, magnetic resonance (MR) based methods offer the possibility to investigate tumor morphology, vasculature, physiology and biochemistry in a single session and have been investigated to get an early insight into tumor response. Several dynamic contrast enhanced MRI (DCE-MRI) studies reported the utility of volume and diameter and the quantitative parameters like volume transfer constant Ktrans, extravascular extracellular volume fraction Ve, flux rate constant kep and blood plasma volume per unit volume of tissue Vp, in the prediction of early response (7–9). Recently, Schaefgen et al. evaluated the predictive ability of mammography, ultrasound and MRI and reported that none of these techniques could diagnose complete pathologic response with good accuracy to replace histological pathological response assessment (10). Apparent diffusion coefficient (ADC) measured using diffusion weighted imaging (DWI) has been reported to be sensitive to changes in the tissue cellularity and emerged as a surrogate biomarker for diagnosis as well as in response assessment (11–13). It was suggested that ADC had the highest specificity to predict the early clinical response compared to the morphological parameters like tumor volume and diameter, however with relatively low sensitivity (11). The change in ADC has been shown to have better correlation with pathological response compared to change in tumor size (14). Minarikova et al investigated the predictive value of segmented tumor volume using DCE-MRI and ADC and demonstrated that the median ADC value was the best predictor of pathological response (15).

Several studies have reported the applications of *in vivo* proton (^1^H) magnetic resonance spectroscopy (MRS) in monitoring the therapeutic response of breast tumors (16–24). These studies documented that prior to therapy; breast tumors showed specific biochemical characteristics like higher concentration of choline containing (tCho) compounds and water content (16–27). Reduction in tCho level and water-to-fat ratio has been reported as probable biomarkers of successful chemotherapy (16–24). Quantitative measurement of tCho concentration showed that changes in tCho occur as early as 24 h after the first cycle of chemotherapy indicating its potential in predicting the early response (20). Danishad et al (21) reported that the sensitivity of tCho resonance signal-to-noise ratio to detect clinical response was 85.7 with 91% specificity while the volume showed 100% sensitivity with 73% specificity. Thus, several MR parameters appear to be candidates as biomarkers for assessing the therapeutic response, although with some limitations in sensitivity and specificity. In recent years several studies have explored the predictive value of combining multi-parameters derived from conventional MRI, DWI, MRS and other methods like FDG-PET to increase the prediction of complete pathological response (15, 28, 29).

The present study was designed to carry out sequential MR measurements in LABC patients with the following objectives: (a) to investigate systematically the changes in tCho, ADC and tumor volume in patients undergoing NACT after I, II, and III cycle of NACT; and (b) to determine the potential clinical utility of multi-parametric approach (combining tCho, ADC and tumor volume) with the pathological and clinical assessments of the tumor response.

## Patients and methods

### Patients

A total of 42 LABC patients (mean age 44.2 ± 10.0 years; range 19–65 years) attending the breast cancer clinic of our Institute and scheduled for NACT were recruited during the period 2007–2016 for this study. Institutional ethical committee approved the study and written informed consent was obtained from each subject.

Patients were evaluated using triple assessment criteria which included clinical history, physical examination and radiological assessment [ultrasonography/mammogram (BIRADS IV and V lesions)] followed by pathology [fine needle aspiration cytology/core biopsy] evaluation. American Joint Committee (AJCC) on cancer tumor, node and metastasis (TNM) staging criteria was followed for the clinical staging. All patients had clinically palpable lumps and the tumor size was measured in two dimensions using Vernier calipers while the tumor volume was calculated using MR images. Details of age, tumor volume, and other relevant parameters are presented in Table 1.

**Table 1:** Clinical and pathological characteristics of forty two LABC patients.

**Characteristics**	**Pathological responders (*n* = 24) (complete and partial)**	**Pathological Non-responders (*n* = 18)**	**Clinical responders (*n* = 26)**	**Clinical non-responders (*n* = 16)**
Mean age (years) (range)	44.7 ± 8.1 (34–65)	43.4 ± 12.3 (19–65)	42.9 ± 9.4 (19–65)	46.3 ± 10.9 (28–65)
**MENOPAUSAL STATUS**				
Pre (*n* = 21)	12	9	15	6
Post (*n* = 21)	12	9	11	10
**T STAGE**				
T2 (*n* = 9)	6	3	5	4
T3 (*n* = 15)	10	5	10	5
T4 (*n* = 18)	8	10	11	7
**ESTROGEN RECEPTOR**				
Positive (*n* = 19)	10	9	12	7
Negative (*n* = 21)	12	9	12	9
NA (*n* = 2)	2	–	2	–
**PROGESTERONE RECEPTOR**				
Positive (*n* = 14)	7	7	8	6
Negative (*n* = 26)	15	11	16	10
NA (*n* = 2)	2	–	2	–
**HER2 RECEPTOR**				
1+ (*n* = 18)	8	10	9	9
2+ (*n* = 4)	4	–	4	–
3+ (*n* = 18)	10	8	11	7
NA (*n* = 2)	2	–	2	–
**CHEMOTHERAPY**				
CAF (*n* = 7)	4	3	6	1
CEF (*n* = 21)	13	8	9	12
DE (*n* = 10)	5	5	8	2
Taxane (*n* = 1)	1	–	1	–
CEF+DE (*n* = 1)	1	–	1	–
DEC (*n* = 1)	–	1	–	1
DE+Herceptin (*n* = 1)	–	1	1	–
**PLANNING AFTER III NACT**				
MRM (*n* = 31)	15	16	17	14
BCS (*n* = 11)	9	2	9	2

*CAF, Cyclophosphamide Adriamycin 5-Fluorouracil (5FU); CEF, Cyclophosphamide Epirubicin and 5FU; DE, Docetaxel Epirubicin; DC, Docetaxel Cisplatin; DEC, Docetaxel Epirubicin Cisplatin; MRM, Modified Radical Mastectomy; BCS, Breast Conservation Surgery (includes wide local excision + axillary clearance); HER2, Human epidermal growth factor; NA, Not Available*.

Study included only those patients with LABC (stage IIA, IIB and IIIA, IIIB) who were not on any hormonal, chemotherapy or radiotherapy prior to the first MR scan. Patients with pregnancy or using contraceptive pills, on prior treatment, claustrophobia, with metallic implants, pacemaker and also unwilling were excluded from the study. Also patients with metastatic disease were excluded as they were treated with a palliative intent.

Metastatic workup of LABC patients was carried out prior to NACT, as per standard guidelines followed at the Institute for complete evaluation of clinical staging of tumor. The workup included liver function tests, chest roentgenograms and ultrasound of the abdomen, pelvis and bone scan. Treatment protocol included NACT followed by surgery and local or loco-regional radiotherapy (Table 1).

### Immunohistochemistry

Biopsied tissues were subjected to histology and immunohistochemical examinations to determine the expression of estrogen receptor (ER), progesterone receptor (PR) and human epidermal growth factor receptor (HER2). Patients with HER2 expression scores of 0 and 1+ were categorized as HER2-negative and those with 3+ were categorized as HER2-positive while those were with 2+ were categorized as equivocal cases. Fluorescent *in-situ* hybridization test could not be done in these patients to determine their accurate HER2 status.

### Assessment of clinical response

Clinical response was determined by measuring the changes in tumor size using Vernier calipers after completion of III NACT. Patients with 50% or more reduction in tumor volume were categorized as clinical responders (cR) while those with no evidence of tumor as complete responders. Patients with less than 50% reduction in tumor volume and/or increase in size were categorized as non-responders (cNR) (11, 30).

### Assessment of pathological response

The volume of the residual tumor after surgery was calculated by an experienced pathologist. To assess the treatment response Miller Payne (MP) grading system was used, and the percentage of cancer cells both in biopsy and post-surgery slides were compared (31). Pathological grades were assigned accordingly and patients showing no change or, no reduction in overall cellularity were categorized as MP grade I. Grade II corresponded to 30% loss of tumor cells while in patients with MP Grade III the estimated tumor cell reduction was found to be in the range of 30–90%. Patients with MP Grade IV corresponded to a loss of more than 90% of tumor cells, while, those with MP Grade V had complete disappearance of malignant cells at the site of tumor with only vascular fibroelastotic stroma seen with macrophages; however, ductal carcinoma *in situ* may be present. We grouped patients with MP grades III and IV as pathological partial responders (pPR). MP grade V as the group showing pathological complete response (pCR), while those with MP grades I & II were categorized as pathological non-responders (pNR) (32). Of the 42 patients examined sequentially, 24 were pathological responders (includes both complete and partial responders) [Grade 3 (*n* = 14); Grade 4 (*n* = 4); Grade 5 (*n* = 6)] while 18 were non-responders [Grade 1 (*n* = 6); Grade 2 (*n* = 12)].

### Criteria for the response assessment using MR parameters

For MR observed tumor volume the same criterion as for clinical response was used (11, 30). For ADC, patients were categorized as responders if their mean ADC after III NACT was higher by 3 times the standard deviation to mean pre-therapy ADC value by using 3 SD criterion described earlier (11). The tCho cut-off value determined after III NACT (clinically; 70.5%, pathologically; 73.1%) was taken to categorize a patient as a responder or a non-responder.

### MR examinations

All MR investigations were carried out at 1.5 T (AVANTO, Siemens Healthcare Sector, Germany) within a week of each chemotherapy cycle. A four channel phased array receive breast matrix was used and patients were positioned prone in coil. After scout images, fat saturated T2-weighted images were obtained in three orthogonal planes using short inversion recovery sequence (TR/TE = 6,940/58 ms; slice thickness = 3–5 mm with no slice gap; matrix size = 320 × 256) to estimate the extent and boundary of tumor. The tumor size in all patients was greater than two centimeters; hence, contrast was not used (19). All MR acquisition parameters, hardware and software used were same during the entire period of this longitudinal study.

Pre-therapy (Tp0) MR examination was performed at least 1 week after core biopsy so that acute edema was settled. A total of 145 MR investigations were carried out on 42 patients. All patients had their pre-therapy MR at Tp0. Thirty eight patients were monitored 1 week after I NACT (Tp1), 24 after II NACT (Tp2) and 41 after III NACT (Tp3).

A single-shot echo planar imaging (EPI) sequence was used to acquire DW images in the transverse plane covering both the breasts to reduce motion artifacts (13). Diffusion gradients were applied along orthogonal directions using: *b* = 0, 500, and 1,000 s/mm^2^; TR/TE = 5,000/87 ms; NS = 1; EPI factor = 128; acquisition matrix = 128 × 128; and slice thickness = 5 mm without any inter slice gap.

Single voxel *in vivo* ^1^H MRS was acquired using reference MR images (PRESS pulse sequence; TR/TE = 1,500/100 ms with 128 averages). Long echo time may result in underestimation of tCho reduction; however, to minimize the effect of fat resonances in breast MRS, a longer TE (≥100 ms) is preferred for improved visibility of tCho signal, despite the loss of signal intensity. A voxel was positioned with appropriate size depending on tumor volume (range 10 × 10 × 10 mm^3^ to 10 × 35 × 45 mm^3^). Both global and manual voxel shimming was carried out (typical line-width of water peak ranging from 8 to 20 Hz). For water suppression, a frequency-selective pre-saturation pulse was used with a bandwidth of 50 Hz. The lipid suppression was achieved using a bandwidth of 1.8 ppm. Using 4–6 saturation bands the outer volume suppression was also carried out. An additional spectrum from the same voxel without water and lipid suppression with the same TE value (100 ms, number of average = 1) was obtained and the internal water signal was used as reference for tCho concentration calculation as described earlier (25). Post-processing was carried out using Syngo GRACE software with a 2.0 Hz line broadening and polynomial order 5 for baseline correction.

### tCho concentration calculation

Using automated normalization with an internal water reference signal, the normalized integral of choline was determined and the absolute tCho concentration was calculated using the formula modified for 1.5 T as described elsewhere (25).

### Tumor volume calculation

The tumor volume was calculated by perimeter method using the formula: volume = ST (A_1_+A_2_+ ….A_n_) where ST is the slice thickness and A_n_ is the area of the tumor of nth slice using the fat suppressed T2-weighted images. All the slices (with no inter slice gap) in which the tumor was seen were used for the volume calculation using free drawn ROIs (RGS and KA). In 6 patients, ROI was drawn twice to find out intra-individual variation, which was verified latter by (US) to check inter-individual variation. The inter-observer agreement was assessed using intra class correlation coefficient, which was 0.99 indicating the better reproducibility of the volume measurements by two different observers.

### ADC calculation

ADC values were calculated based on monoexponential fitting method. Contiguous circular ROIs of five pixels were drawn (diameter range: from 0.09 to 0.12 cm^2^) on the hypo-intense areas of the tumor on the parametric image of ADC map (11). The average ADC from all such ROIs from the tumor was reported. The number of ROIs used for the calculation of mean ADC prior to therapy varied among patients depending on the tumor size.

### Data analyses

A retrospective analysis for comparing tCho, ADC and tumor volume during various cycles of NACT with the clinical and the pathological response status was carried out. Statistical analyses were carried out using statistical software SPSS 16 and STATA 9. A generalized estimating equation (GEE) was used to compare the tCho concentration, mean ADC and tumor volume following NACT. Receiver operating characteristic curve (ROC) analysis was carried out to determine the cut-off percentage change of tCho, ADC and volume to differentiate between both clinical and pathological responders and non-responders after I, II and III NACT. The optimum cut-off value was chosen as a particular point on the ROC curve, where sensitivity was equal to specificity. The cut-off percentage obtained for tCho after III NACT was used to categorize patients as responders or non-responders, in the absence of any literature data. Fisher exact test was carried out to analyze the significance of clinical categorical variables HER2 status, ER and PR, and tumor stage in the prediction of tumor response. The agreement between clinical and pathological responses was carried out using weighted kappa statistics.

The normalized value of tCho, ADC and volume after each cycle of NACT to their pre-therapy value was also calculated. Statistical models from principal component analysis (PCA) were built using the normalized values of MR parameters to explore any clustering behavior of responder and non-responder patients using MetaboAnalyst 4.0 web server (www.metaboanalyst.ca). Analysis of component plots was used to identify similarities and differences between the categories (pR vs. pNR; cR vs. cNR).

## Results

**Figure 1:**
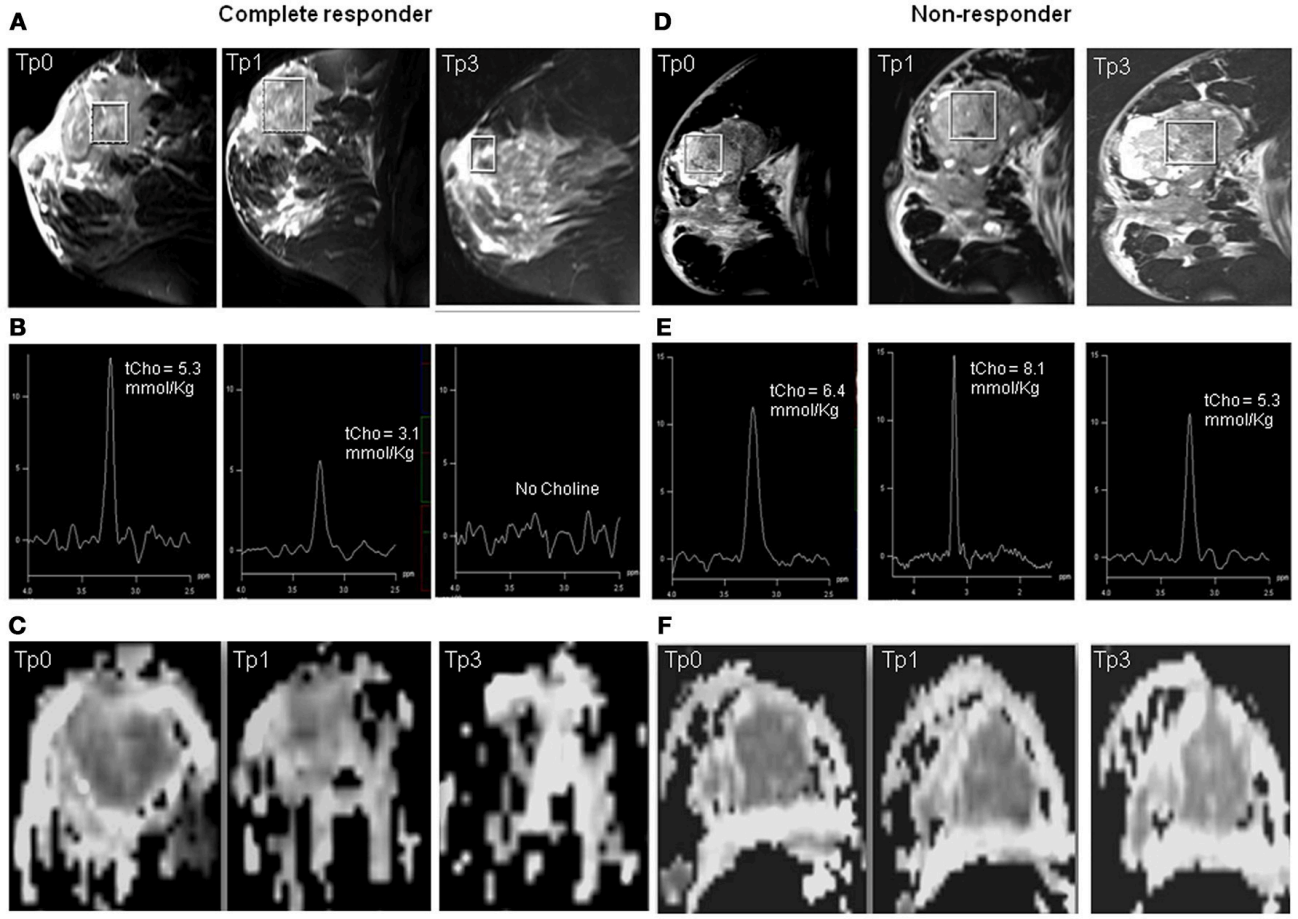
Representative example of T2 weighted sagittal MR images **(A)** showing the voxel location and the corresponding proton MR spectra acquired **(B)** from the voxel shown in **(A)** of a patient who showed complete response both pathologically and clinically, acquired at Tp0, Tp1, and Tp3 while **(C)** shows the corresponding ADC maps. The representative example of a patient who was a non-responder both pathologically and clinically: **(D)** T2 weighted sagittal MR images showing the voxel location and **(E)** the corresponding proton MR spectra, and **(F)** the corresponding ADC maps acquired at Tp0, Tp1, and Tp3.

Of the 42 patients examined sequentially, 24 were pathological responders while 18 were non-responders. On clinical evaluation, 26 were responders and 16 non-responders (includes patients with clinically stable or progressive disease). Figure 1 shows the representative example of T2 weighted sagittal MR images (A) showing the voxel location and the corresponding proton MR spectra (B) acquired from the voxel shown in (A) of a patient who showed complete response both pathologically and clinically, acquired at Tp0, Tp1, and Tp3; while (C) shows the corresponding ADC maps. Figures 1D–F shows the T2-weighted images, the corresponding proton MR spectra and the ADC maps of a patient who was a non-responder both pathologically and clinically acquired at Tp0, Tp1, and Tp3.

### Clinical characteristics and response status

The percentage of patients showing positive clinical and pathological responses to chemotherapy did not differ significantly among T2, T3, and T4 stage tumors. The percentage of clinical responders were higher with estrogen receptor positive and progesterone receptor negative status, while patients with estrogen receptor negative and progesterone receptor negative statuses showed higher percentage of pathological response (Table 1). Human epidermal growth factor (HER2+) patients showed a better response (clinical, 61.1%, pathological, 55.6%) as compared to those with a HER2- (clinical, 50.0%, pathological, 44.4%) receptor status. Also, those with a HER2 2+ status showed 100% response both clinically and pathologically. Pre-menopausal women (71.4%) showed higher percentage of clinical response compared to post-menopausal women (52.4%), while a pathological response rate of 57.1% was achieved for patients with both menopausal statuses (Table 1). Fischer Exact test showed none of the parameters as a significant predictor of response.

### Pathological response with MR parameters

**Figure 2:**
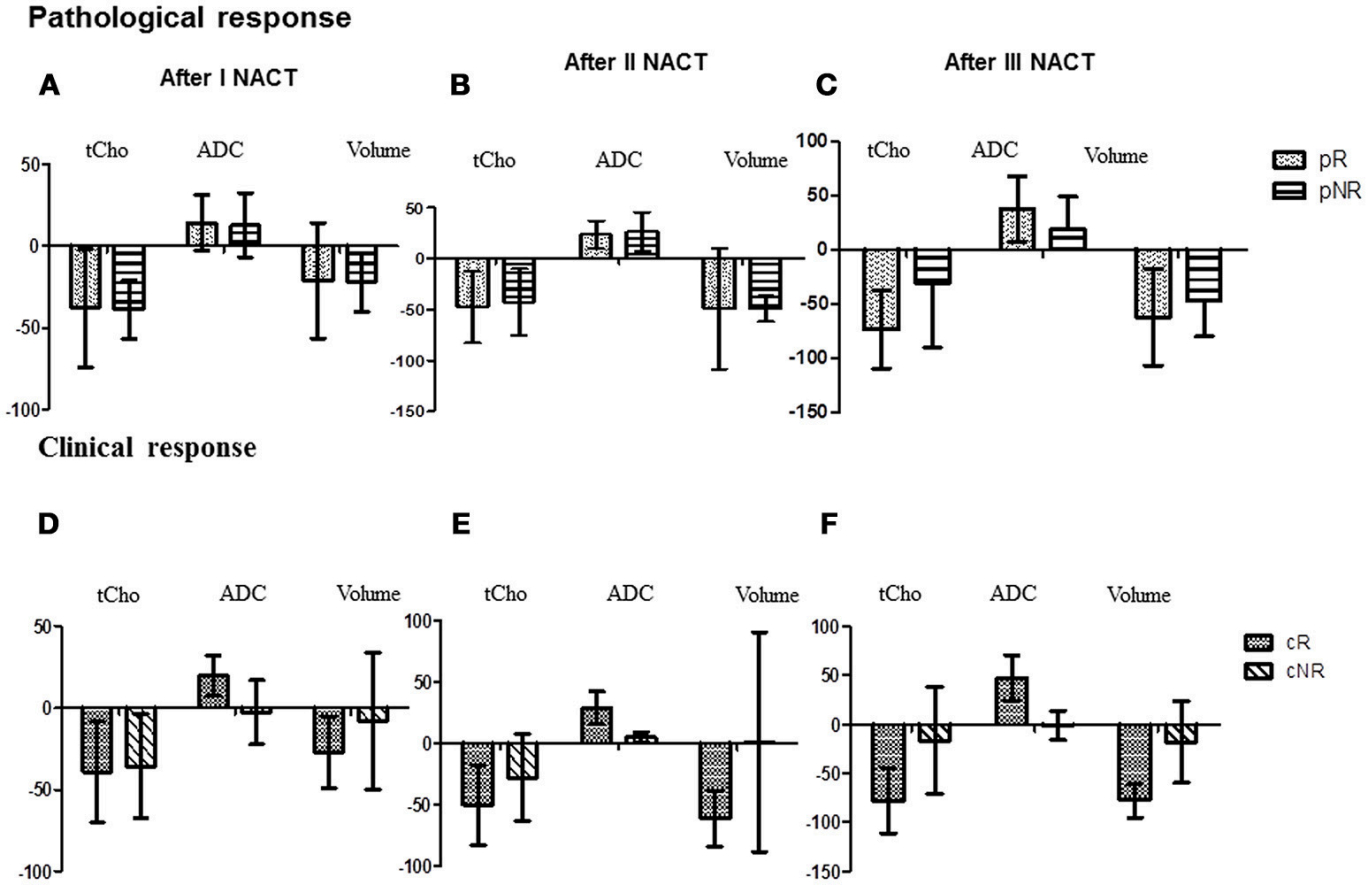
The bar diagram showing the percentage changes in three MR parameters (tCho, ADC and volume) in pathological responders and non-responder after I **(A)**, II **(B)**, and III NACT **(C)** compared to the pre-therapy value. While **(D–F)** show percentage changes in these parameters in clinical responder and non-responder patients.

tCho at Tp0 was significantly higher in pR compared to pNR and it reduced significantly at Tp1 followed by a gradual decrease at Tp2 and Tp3, however, the percentage reduction was lower in pR (see Table 2 and Figures 2A–C). At Tp0, ADC was similar for pR and pNR and at Tp3 it showed significant increase in both pR and pNR with % increase being higher in pR (Table 2 and Figure 2). Tumor volume at Tp0 was lower in pR compared to pNR and it reduced significantly at Tp2 and Tp3 in pR while in pNR, the reduction was seen only at Tp3 with the percentage decrease being lower compared to pR (Table 2).

**Table 2:** Comparison of the concentration of total choline (tCho), apparent diffusion coefficient (ADC) and volume in both pathological and clinical responders (R) and non-responders (NR) at pre-therapy (Tp0) and after I (Tp1), II (Tp2), and III (Tp3) NACT.

**Time point**	**tCho (mM/Kg)**		**ADC (10**^**−3**^ **mm**^**2**^**/s)**		**Volume (cm**^**3**^**)**	
	**pR**	**pNR**	**pR**	**pNR**	**pR**	**pNR**
**PATHOLOGICAL RESPONSE**						
						
Tp0	5.75 ± 3.44^*^^#^^@^^$^ (*n* = 24)	4.29 ± 2.15^*^^#^^@^^$^ (*n* = 18)	1.00 ± 0.16^*^^#^^@^ (*n* = 24)	1.05 ± 0.15^#^^@^ (*n* = 18)	74.15 ± 61.65^#^^@^^$^ (*n* = 24)	116.36 ± 93.92^@^^$^ (*n* = 18)
Tp1	3.00 ± 2.21^*^ (*n* = 22)	3.09 ± 1.46^*^ (*n* = 12)	1.12 ± 0.16^*^ (*n* = 22)	1.10 ± 0.13 (*n* = 13)	53.40 ± 45.56 (*n* = 22)	80.06 ± 55.14 (*n* = 13)
Tp2	2.28 ± 2.08^#^ (*n* = 17)	2.90 ± 1.52^#^ (*n* = 8)	1.19 ± 0.14^#^ (*n* = 17)	1.20 ± 0.20^#^ (*n* = 8)	29.32 ± 22.12^#^ (*n* = 17)	51.42 ± 42.96 (*n* = 8)
Tp3	1.46 ± 2.12^@^ (*n* = 24)	2.63 ± 1.93^@^ (*n* = 17)	1.35 ± 0.20^@^ (*n* = 24)	1.19 ± 0.20^@^ (*n* = 17)	21.61 ± 18.59^@^ (*n* = 24)	53.77 ± 50.27^@^ (*n* = 17)
	**cR**	**cNR**	**cR**	**cNR**	**cR**	**cNR**
**CLINICAL RESPONSE**						
Tp0	5.47 ± 2.94^*^^#^^@^ (*n* = 26)	4.56 ± 3.15 (*n* = 16)	0.96 ± 0.13^*^^#^^@^^$^ (*n* = 26)	1.12 ± 0.16^$^ (*n* = 16)	90.90 ± 82.47^#^^@^ (*n* = 26)	94.42 ± 75.51 (*n* = 16)
Tp1	2.99 ± 1.70^*^ (*n* = 25)	3.14 ± 2.66 (*n* = 9)	1.14 ± 0.14^*^ (*n* = 25)	1.04 ± 0.14 (*n* = 10)	60.08 ± 50.52 (*n* = 25)	71.37 ± 51.35 (*n* = 10)
Tp2	2.04 ± 1.32^#^ (*n* = 20)	4.23 ± 2.96 (*n* = 5)	1.21 ± 0.13^#^ (*n* = 20)	1.07 ± 0.06 (*n* = 4)	28.35 ± 21.33^#^ (*n* = 20)	68.56 ± 45.54 (*n* = 5)
Tp3	1.02 ± 1.40^@^ (*n* = 26)	3.55 ± 2.18 (*n* = 15)	1.40 ± 0.15^@^ (*n* = 26)	1.08 ± 0.13 (*n* = 15)	16.12 ± 15.69^@^ (*n* = 26)	67.57 ± 44.1 (*n* = 15)

* *Denotes significance between values at pre therapy and I NACT*.

# *Denotes significance between values at pre therapy and II NACT*.

@ *Dnotes significance between values at pre therapy and III NACT*.

$ *Denotes significance between values at pre therapy*.

Receiver operating characteristic curve (ROC) analysis was used to determine the cut-off value (% change) of MR parameters to differentiate between the pR and pNR at Tp1 and Tp3 (Table 3). Agreement test using Kappa statistics between various MR parameters and the pathological response were also worked out (Table 4). The value of k was 0.38 for tumor volume and tCho indicating a fair agreement with the pathological response. ADC and the multi-parametric approach showed no agreement with the pathological response.

**Table 3:** Cut-off values of MR parameters (% change in tCho, ADC and volume) to differentiate pathological and clinical responders and non-responders using ROC analysis.

**Time points**	**tCho (mm/Kg)**	**ADC (10^−3^ mm^2^/s)**	**Volume (cm^3^)**
**CUT-OFF VALUES TO DIFFERENTIATE PATHOLOGICAL RESPONDERS AND NON-RESPONDERS**			
Tp1	Cut-off (−39.43%) (Sens: 66.7%; Spec: 63.6%; AUC: 0.54)	Cut-off (13.19%) (Sens: 50%; Spec: 50%; AUC: 0.49)	Cut-off (−21.33%) (Sens: 53.8%; Spec: 50%; AUC: 0.52)
Tp3	Cut-off (−73.13%) (Sens: 70.6%; Spec: 66.7%; AUC: 0.76)	Cut-off (28.32%) (Sens: 66.7%; Spec: 64.7%; AUC: 0.69)	Cut-off (−64.53%) (Sens: 70.6%; Spec: 66.7%; AUC: 0.68)
**CUT-OFF VALUES TO DIFFERENTIATE CLINICAL RESPONDERS (Cr) AND NON-RESPONDERS (CNR)**			
Tp1	Cut-off (−39.80%) (Sens: 55.6%; Spec: 52%; AUC: 0.52)	Cut-off (10.72%) (Sens: 92%; Spec: 90%; AUC: 0.89)	Cut-off (−16.49%) (Sens: 60%; Spec: 68%; AUC: 0.69)
Tp3	Cut-off (−70.51%) (Sens: 80%; Spec: 76.9%; AUC: 0.88)	Cut-off (25.50%) (Sens: 88.5%; Spec: 100%; AUC: 0.97)	Cut-off (−56.83%) (Sens: 93.3%; Spec: 88.5%; AUC: 0.97)

*AUC, Area under the curve; Sens, sensitivity; Spec, specificity*.

**Table 4:** Kappa results between various MR parameters, clinical and pathological response.

**Parameter vs. parameter**	**Kappa**	***p*-value**
tCho vs. pathological response	0.31	0.05
ADC vs. pathological response	0.18	0.19
Tumor volume vs. pathological response	0.38	0.01
Multi-parametric vs. pathological response	0.25	0.11
tCho vs. clinical response	0.49	0.002
ADC vs. clinical response	0.47	< 0.001
Tumor volume vs. clinical response	0.78	< 0.001
Multi-parametric vs. clinical response	0.80	< 0.001
Pathological vs. clinical Response	0.41	0.008

**Figure 3:**
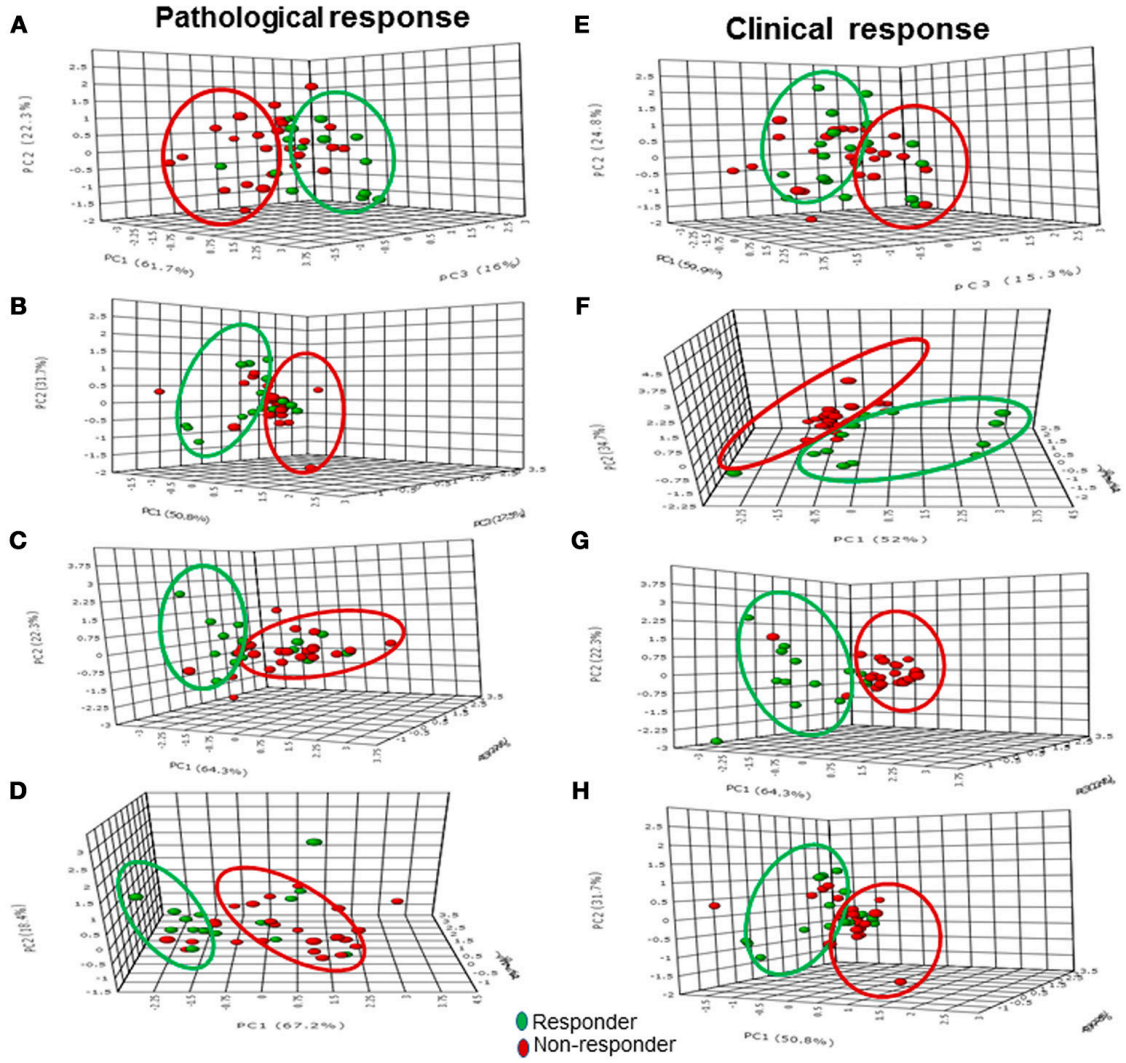
The 3-D score plot (PC1-PC3) of PCA analysis of multi-parametric data (volume, ADC and tCho) in pathological responders and non-responders at Tp0 **(A)** after Tp1 **(B)**, Tp2 **(C)**, and Tp3 **(D)**, while **(E–H)** show the 3-D score plot for clinical response.

Sensitivity and specificity values for individual MR parameters and multi-parametric approach for the detection of pathological response (Table 5) showed that volume had a higher sensitivity (83.3%) while ADC showed a higher specificity (76.5%). Combination of all three MR parameters resulted in 66.7% sensitivity with 58.8% specificity. Further multivariate PCA using pretherapy values of three MR parameters (tCho, ADC, volume) demonstrated clustering pattern between pR and pNR using principal components 1 to 3, however, with some overlap (Figure 3A).

**Table 5:** Response assessment using individual MR biomarkers (tCho concentration, ADC and volume) and in combination using pathological and clinical response as gold standards for patients monitored at Tp3.

	**Pathological response**		**Clinical response**	
**tCho**				
	R	NR	R	NR
R	16	6	20	4
NR	8	11	6	11
Sens: 66.7%; Spec: 64.7%; Acc: 65.9%; PPV: 72.7%; NPV: 57.9 %			Sens: 76.9%; Spec: 73.3%; Acc: 75.6%; PPV: 83.3%; NPV:64.7%	
**ADC**				
	R	NR	R	NR
R	10	4	14	0
NR	14	13	12	15
Sens: 41.7%; Spec: 76.5%; Acc: 56.1%; PPV: 71.4%; NPV:42.2%			Sens: 53.9%; Spec: 100%; Acc: 70.7%; PPV: 100%; NPV: 55.6%	
**Volume**				
	R	NR	R	NR
R	20	8	25	3
NR	4	9	1	12
Sens: 83.3%; Spec: 52.9%; Acc: 70.7%; PPV: 71.4%; NPV:69.2%			Sens: 96.2%; Spec: 80.0%; Acc: 90.2%; PPV: 89.3%; NPV: 92.3%
**Multiparametric-tCho-ADC-volume**				
	R	NR	R	NR
R	16	7	22	0
NR	8	10	4	15
Sens: 66.7%; Spec: 58.8%; Acc: 63.4%; PPV: 70.0%; NPV: 55.6%			Sens: 84.6%; Spec: 100%; Acc: 90.2%;PPV: 100%; NPV: 79%	

*Sens, sensitivity; Spec, specificity; Acc, Accuracy; PPV, positive predictive value; NPV, negative predictive value; R, Response; NR, No-response*.

As mentioned earlier, normalized values of tCho, ADC and volume after each cycle of NACT to their pre-therapy value were also calculated. PCA model built using normalized values of MR parameters (tCho, ADC, volume) clearly demonstrated separate clusters of pR and pNR based on principal components 1 to 3 (Figures 3B–D) at Tp1, Tp2, and Tp3.

### Clinical response with MR parameters

A significant early reduction was seen in tCho at Tp1 followed by a gradual reduction at Tp2 and Tp3 in cR while there was no change in cNR group following NACT (Table 2 and Figures 2D–F). At baseline, ADC was significantly lower in cR than in cNR and the value increased at Tp1, Tp2, and Tp3 (Table 2 and Figures 2D–F). However, no significant change was seen in cNR following NACT.

Tumor volume significantly decreased at Tp2 and Tp3 compared to its value at Tp0 in cR, while cNR showed no significant difference during NACT (Table 2). Changes were seen in these biomarkers at various time points from Tp1 to Tp3 (Figures 2D–F). The cut-off of percentage of tCho, ADC and volume to differentiate between cR and cNR after I and III NACT using ROC were done (Table 3).

Kappa statistics between various MR parameters and the clinical response showed a *k* value of 0.78 for tumor volume showing substantial agreement with the clinical response, while tCho and ADC showed only moderate agreement (Table 4). Multi-parametric MR showed substantial agreement (*k* = 0.80) with the clinical response.

Sensitivity and specificity for individual MR parameters and multi-parametric approach for detection of clinical response showed that volume had a higher sensitivity (96.2%) while ADC showed a higher specificity (100%) for the detection of clinical response (Table 5). Combination of all three MR parameters resulted in 84.6% sensitivity with 100% specificity. PCA using pretherapy values of three MR parameters (tCho, ADC, volume) demonstrated clustering pattern of pR and pNR based on principal components 1 to 3, however, with some overlap (Figure 3E). PCA using normalized values of MR parameters (tCho, ADC, volume) clearly demonstrated separate clusters of cR and cNR (Figures 3F–H).

### Correlation between pathological and clinical responses

In the present study, a moderate agreement (*k* = 0.46, *p* = 0.003) between clinical and histological response prediction was observed (Table 4).

## Discussion

The response to NACT is highly variable in individuals indicating the need for patient tailored treatment regimes for improved outcome and survival. It has been reported that nearly 70% patients with breast cancer demonstrated a clinical response to NACT on physical examination or radiological imaging methods, however, only 3–40% of them achieve a pathological complete response (33). Identification of non-responders at an early stage of NACT would facilitate treatment management and in taking decisions like change of therapy or surgery. In this regard, non-invasive biomarkers that can predict treatment response either prior to the onset of treatment or early in the course of treatment would be of great clinical benefit. Therefore in the present study, the use of three MR biomarkers (tCho, ADC, and MR tumor volume) individually and their combination (multi-parametric approach), has been evaluated in the assessment of early therapeutic response (both pathological and clinical).

Firstly we discuss the correlation of pathological response estimate with the MR response. Statistically significant reduction in tCho concentration was seen as early as at Tp1 with complete disappearance of tCho peak in 7/24 in pathological responders after III NACT. This finding was in agreement with the literature and the reduction was attributed to the inhibition of cellular proliferation and the cytotoxic effect of chemotherapy (20, 23, 24, 26, 30). It has been reported that NACT increases apoptosis within 24 h after the initiation of therapy in breast cancer (34, 35).

An early change was also seen in ADC compared to baseline value at Tp1 in pR. The value of ADC also increased in pNR after Tp2 and Tp3 with the percentage increase being lower than pR. These changes were in agreement with the previous studies reported in the literature (11, 12, 36). The increased ADC was attributed to the chemotherapy-induced cell apoptosis which led to decreased cellularity in breast cancer (34, 35, 37–39).

Tumor volume reduced significantly only after II and III NACT in pR. Reduction in tumor volume was also seen in pNR but only after III NACT with the % decrease being lesser than pR. Furthermore in concordance with the literature (40), pNR showed a significantly larger tumor volume at baseline compared to pR. It was reported that due to changes in vascularity, distribution of nutrients might not be consistent in growing tumors of large size (41). Thus on the surface regions of a large sized tumor, i.e., in the outer regions, cells might receive sufficient nutrients and cell proliferation continues. In the middle regions, the nutrient supply could be sufficient enough only to maintain the viability of cells (41) while central regions could be deprived of nutrients resulting in cell death and formation of necrotic cores (41). This non-uniform vasculature might have affected the distribution of nutrients as well as the availability of chemotherapeutic drugs at the tumor inner core and thus leading to non-response in large sized tumors as seen in pNR group. On the contrary in pR patients, a relatively faster uptake of nutrients and the chemotherapy drugs by viable and actively proliferating cells might have contributed to positive response to chemotherapy. Studies have documented the differences in treatment response that was related to the amount of necrosis in a given tumor and the fact that necrotic tumors often were hypoxic, acidotic and poorly perfused (42, 43). All these factors could explain the resistance to treatment seen in our patient cohort.

This non-uniform distribution of nutrients in large sized tumors was also reflected in the tCho levels, which was higher at baseline in pR than in pNR and was in agreement with the earlier studies (23, 24). This suggested that relatively small sized tumors with better nutrient supply and metabolic activity demonstrated positive pathological response to chemotherapeutic drugs. Interestingly, PCA score plot using combination of all three MR parameters at Tp0 demonstrated a clustering pattern between pR and pNR though with some overlap indicating that pre-therapy MR parameters may probably have the potential of differentiating between the two groups. Meisamy et al. (20) reported higher tCho levels in responders compared to non-responders which were also in concordance with our study. Few studies on breast tumors (37, 38) reported a strong negative correlation between pretreatment tumor ADC and tumor size reduction during therapy.

Further, our results revealed that tumor volume showed higher sensitivity (83.3%) in comparison to sensitivities of tCho (66.7%) and ADC (41.7%) to detect pathological response. The specificity to differentiate pNR was higher for ADC, the values being 76.5, 64.7, and 52.9% for the ADC, tCho and volume, respectively. Further, it was seen that combination of all three MR parameters showed 66.7% sensitivity and 58.8% specificity. Kappa statistics demonstrated that among the three MR parameters, Further our results showed that tumor volume (*k* = 0.38) and tCho (*k* = 0.31) showed fair agreement with the pathological response while ADC (*k* = 0.18) showed only slight agreement. The multi-parametric approach also resulted in the low kappa coefficient (0.25) indicating slight agreement. The statistical models built from PCA using normalized values of MR parameters (tCho, ADC, volume) clearly demonstrated the separate clusters of pR and pNR.

We further assessed the diagnostic performance of these 3 MR parameters individually and in combination (multi-parametric approach) with clinical response. The value of tCho reduced significantly as early as I NACT in cR while in cNR no significant change was seen during the course of three cycles of NACT. Similarly, an increased ADC was observed at Tp1 while its value remained same in cNR during the course of the therapy. Tumor volume showed significant reduction only at Tp2 in cR while no change was seen in cNR. The sensitivity to detect clinical response was highest for tumor volume (96.2%) in comparison to the sensitivities of the parameters, tCho (76.9%) and ADC (53.9%). The specificity to differentiate cNR was higher for ADC (100%) while it was 73.3 and 80.0% for tCho and volume, respectively. Further, it was seen that combination of all three MR parameters showed 84.6% sensitivity and 100% specificity. Interestingly, PCA score plot using combination of all three MR parameters at Tp0 demonstrated a clear clustering pattern between cR and cNR in contrast to the plot of pR and pNR. This finding further substantiated the role of pre-therapy MR parameters in predicting response.

Moreover our data showed that tumor volume (*k* = 0.78) and multi-parametric approach (*k* = 0.80) showed substantial while tCho (*k* = 0.49) and ADC (0.47) only moderate agreement with the clinical response. Thus our data indicated that diagnostic performance of multi-parametric approach as well as of individual parameters was better for the prediction of clinical response as compared to pathological response. The statistical models from PCA built using normalized values of MR parameters (tCho, ADC, volume) clearly demonstrated separate clusters of cR and cNR. Further, clinical response showed a moderate agreement (*k* = 0.41, *p* = 0.008) with histological response in the present study. Further, cut-off values of percentage change in individual parameters (tCho, ADC and volume) to discriminate between pR vs. PNR and cR vs. cNR were also calculated for the future studies using ROC analysis.

As discussed earlier, response assessment is an essential component of patient management and Feldman et al. (44) have reported that 45% of patients with clinically complete response after treatment had gross macroscopic tumor while 60% of patients with no gross macroscopic tumor were found to have residual tumor on clinical examination. In the present study 15.4% of patient with clinical response had some residual tumor on histology due to the presence of residual fibrosis and indistinct tumor margins. Thus clinical examination alone was reported to be insufficient for response assessment and prediction of residual tumor size (45, 46). However, clinical response has its own relevance in actual clinical scenario as this is the first line of patient management. Though, pathological response is the gold standard to find out accurately the response status and also whether any residual tumor cells are present. However it can be assessed only after surgery that is planned after 3 or 6 cycles of NACT.

Recently, Bouzan et al. reported that sensitivity and specificity of MRI for diagnosing invasive residual disease was 75 and 78.5% (8). They also reported that the accuracy of MRI in estimating the residual disease varies with the tumor grade and hormonal receptor status (8). Further, chemotherapy induced changes like cell death and formation of necrotic and fibrotic areas increases the heterogeneity of the large sized tumors which would affect the quantitative assessment of MR functional parameters like ADC and tCho concentration. Fujimoto et al. (14) reported that the change in ADC after chemotherapy had better correlation (*r* = 0.67) than the change in tumor size (*r* = 0.58) after NACT with pathological response which was in contrast to our findings. A pooled analysis that included 15 DWI studies demonstrated 88% sensitivity and 79% specificity for prediction of pR which was higher than reported in the present study (47). However, their analysis reported variety of issues related to DWI data and suggested the need for well-designed clinical trials for assessing the utility of DWI in predicting pR (47). Very recently a study by Adoui et al has explored the use of parametric response map method that analyzed voxel-by-voxel temporal changes after I NACT using MRI (48). The diagnostic accuracy of tCho quantification (49) and DCE-MRI (50) was reported to be more sensitive for prediction of pathological response in triple negative cancer. In a multi-site clinical trial setting for evaluating the potential of ^1^H MRS in predicting early chemotherapy response, technical difficulty of acquiring quantitative MRS were reported as a major challenge (51). Thus, large variability in the data across the studies necessitates further optimization of acquisition and quantitation methods.

Several studies in recent years have focused on multi-parametric and multimodality approach to develop a strategy to arrive at a fairly high predictive value of pathological response. However, it appears to be a long path to achieve this goal and better designed studies are essential. Our present data has provided information which would be useful for further improvement in treatment response assessment. However, the study needs to be conducted on a large cohort of patients so that the technique could be used for breast cancer management in clinical settings.

This study has few limitations. Firstly, only conventional MRI was used for tumor volume measurements rather than DCE-MRI. However, a recent study reported that unenhanced MRI gave similar results to DCE-MRI for the tumor assessment to NACT (9). Thus, our results demonstrated that conventional MRI methods without contrast may be sufficient for pathological response prediction. Secondly the less number of patients studied longitudinally in this study. More number of patients as well as more centers needs to carry out such studies to arrive at a possible early predictor of response non-invasively.

## Conclusions

Present study evaluated the utility of three MR parameters (tCho, ADC and volume) individually and also in combination (multi-parametric MR approach) in predicting both pathological and clinical response in patients with LABC undergoing NACT treatment. The data suggested that the MR parameters, ADC and tCho exhibited changes at pre-therapy values and as early as I NACT while reduction in tumor volume was seen only after 2nd cycle, both in pathological and clinical responders. This indicated that in predicting early response, tCho and ADC may have substantial potential in comparison to volume measurements. After 3rd cycle of NACT, MR volume showed higher sensitivity in comparison to tCho, ADC and multi-parametric approach for the prediction of pathological response. Similarly for the prediction of clinical response, MR volume showed highest sensitivity while increase in specificity was seen with the multi-parametric approach. ADC exhibited highest specificity in differentiating both pathological and clinical responder patients. After 3rd cycle of chemotherapy, both MR volume and multi-parametric approach, demonstrated substantial agreement while tCho and ADC showed only moderate agreement. MR parameters exhibited lesser agreement with the pathological response (moderate with tumor volume, fair with tCho and slight agreement with ADC) in comparison to the clinical response. Pathological and clinical responses showed moderate agreement.

Interestingly, multivariate analysis using combination of all three MR parameters demonstrated a clustering pattern between (pR vs. pNR; cR vs. cNR) though with some overlap indicating that pre-therapy MR parameters may probably have the potential in differentiating the two groups. After 3rd cycle of NACT, the multivariate PCA model showed separate clusters for pR vs. pNR and cR vs. cNR.

In conclusion our present study of evaluating the correlation of both the clinical and the pathological responses with the MR data in the same set of patients demonstrated that multi-parametric approach achieved 100% specificity in predicting the clinical response while MR volume emerged as a highly suitable predictor for both clinical and pathological assessments after III NACT. Further, this study demonstrated that MR parameters while used individually and in combination have the potential of being used as non-invasive predictors of pathological as well as clinical responses. Future study needs to be carried out in multi-centers in a large cohort of patients for further improvements in breast cancer management.

## Author contributions

NJ and US conceived the hypothesis and NJ, US, RP, and VS designed the experiments. RP and VS recruited patients and carried out clinical work-up while SM and SG carried out histopathological evaluation. RS and KA performed MR experiments. RS, US, KA, and NJ analyzed and interpreted the data. US, KA, and NJ wrote the manuscript that was reviewed by all authors and approved.

### Conflict of interest statement

The authors declare that the research was conducted in the absence of any commercial or financial relationships that could be construed as a potential conflict of interest.

## Abbreviations

MRI: Magnetic resonance imaging
DCE-MRI: Dynamic contrast enhanced MRI
BCS: Breast conservation surgery
DWI: Diffusion weighted Imaging
MRS: Magnetic resonance spectroscopy
ADC: Apparent diffusion coefficient
tCho: Total choline
W-F: Water-to-fat ratio
LABC: Locally advanced breast cancer
NACT: Neoadjuvant chemotherapy
ER: Estrogen receptor
PR: Progesterone receptor
HER: Human epidermal growth factor receptor
AJCC: American joint cancer committee
TNM, Tumor: node and metastasis
cR: Clinical complete responder
cNR: Clinical non-responder
pPR: Pathological partial responder
pCR: Pathological complete responder
pNR: Pathological non-responder
ROI: Region of interest
TR: Repetition time
TE: Echo time
STIR: Short tau inversion recovery
EPI: Echo planar imaging
PET: Positron emission tomography
SPECT: Single photon emission computed tomography
GEE: General estimated equation
ROC: Receiver operating characteristic curve
MP: Miller payne grading
Sens: Sensitivity
Spec: Specificity
AUC: Area under curve
R: Response
NR: No-response.
